# First isolation of three *Orientia tsutsugamushi* strains (Boryong, Kawasaki, and Yonchon) from trombiculid mites in the Republic of Korea

**DOI:** 10.3389/fcimb.2026.1994382

**Published:** 2026-10-23

**Authors:** Hyunyoung Yoon, Byoungchul Gill, Jin Seo Park, Hak Seon Lee, Seong Yoon Kim, Hee Il Lee

**Affiliations:** 1 Division of Vectors and Parasitic Diseases, Korea Disease Control and Prevention Agency, Cheongju, Republic of Korea; 2 Infectious Disease Inspection Division, Animal and Plant Quarantine Agency, Seoul, Republic of Korea; 3 Division of Bacterial Diseases, Korea Disease Control and Prevention Agency, Cheongju, Republic of Korea; 4 Department of Veterinary Medicine & Public Health, Armed Forces Medical Research Institute, Daejeon, Republic of Korea

**Keywords:** *Orientia tsutsugamushi*, vector-derived isolation, trombiculid mites, scrub typhus, NOD.SCID mice, molecular epidemiology

## Abstract

Scrub typhus is an acute febrile illness caused by *Orientia tsutsugamushi*, an obligate intracellular bacterium transmitted by trombiculid mites. Although numerous *O. tsutsugamushi* strains have been identified in human patients, recovery of viable strains from trombiculid mites remains technically challenging. In this study, we report the successful isolation and stable establishment of three *O. tsutsugamushi* strains, namely, Boryong, Kawasaki, and Yonchon, directly from field-collected trombiculid mites in the Republic of Korea (ROK). Trombiculid mites collected from wild rodents at four regions (Cheorwon, Cheongju, Geoje, and Hapcheon) in November 2025 were surface-sterilized, homogenized, and screened for *O. tsutsugamushi*. Positive homogenates were inoculated intraperitoneally into immunodeficient NOD.SCID mice to facilitate *in vivo* amplification. Spleen homogenates from infected mice collected upon the onset of clinical signs were subsequently propagated in L929 cell cultures. Successful isolation was confirmed by cytopathic effects-like morphological changes, immunofluorescence assay, real-time quantitative PCR, nested PCR, and sequencing of the 56-kDa *tsa* and 47-kDa *htrA* genes. Phylogenetic analyses demonstrated that the established isolates clustered within the known Boryong, Kawasaki, and Yonchon lineages. All isolates were stably maintained through serial passages *in vitro*, exhibited robust intracellular replication, and were free of co-infection with other representative tick- and trombiculid-borne intracellular bacteria. This study provides direct evidence that viable *O. tsutsugamushi* strains are maintained within natural trombiculid mite populations and can be successfully isolated using a combined *in vivo*–*in vitro* approach, representing the first successful isolation of mite-derived strains in the ROK and the first recovery of Boryong, Kawasaki, and Yonchon strains from natural mite hosts. These vector-derived isolates represent valuable biological resources for future studies on the ecology, molecular epidemiology, pathogenesis, and prevention of scrub typhus.

## 1 Introduction

Scrub typhus is an acute febrile illness caused by the obligate intracellular bacterium *Orientia tsutsugamushi*, which is transmitted to humans through the bite of infected larval trombiculid mites (Lim and Wang, 2018). In the Republic of Korea (ROK), it remains a significant public health concern, with approximately 4,000 cases reported annually and a pronounced seasonal peak during autumn, particularly in October and November (Jeong et al., 2023). The disease has traditionally been considered endemic to the Asia-Pacific region, including East and Southeast Asia (Xu et al., 2017). However, the identification of novel *Orientia* species, such as *Candidatus Orientia chiloensis* and *Orientia chuto*, together with increasing reports from regions outside the traditional endemic zone, including South America and the Middle East, suggests that the geographic distribution of *Orientia* species may be broader than previously recognized (Abarca et al., 2020). Because the clinical manifestations of scrub typhus are often nonspecific during the early stage of infection, the disease can be difficult to distinguish from other acute febrile illnesses, potentially delaying appropriate diagnosis and treatment. Such delays may allow disease progression and increase the risk of severe complications, including sepsis, respiratory failure, and cardiac dysfunction (Baranwal and Subramani, 2019; Sivasubramanian et al., 2024; Tarale and Sajjanar, 2024). These epidemiological and clinical characteristics underscore the importance of isolating and characterizing circulating *O. tsutsugamushi* strains to improve our understanding of their genetic diversity and regional distribution.

*Orientia tsutsugamushi* is characterized by considerable genetic and antigenic diversity, with variations in the 56-kDa *tsa* gene serving as a key marker for strain differentiation (Varghese et al., 2015; Luce-Fedrow et al., 2018). This diversity has significant implications for disease progression, host immune response, diagnostic accuracy, and vaccine development (Ha et al., 2015; Kala et al., 2020). Furthermore, antigenic variation among strains can influence the sensitivity and specificity of current diagnostic assays; moreover, some strains have been reported to limit the efficacy of existing vaccine candidates (Luce-Fedrow et al., 2018; Yang et al., 2019). Therefore, precise characterization of strain diversity is essential not only for clinical management but also for the design of effective preventive strategies.

To date, numerous strains have been isolated and reported from human patients, contributing to an increasing understanding of the molecular epidemiology of scrub typhus (Yang et al., 2012; Wongprompitak et al., 2015). In contrast, recovery of *O. tsutsugamushi* from trombiculid mites, the primary biological vector and natural reservoir of the pathogen, has been reported only rarely (Tamura et al., 2000; Urakami et al., 2000; Elliott et al., 2019). Isolation from mites is technically challenging due to their small size (~150–300 µm) (Alexander et al., 2024), low bacterial loads, and the strict intracellular growth requirements of the organism (Elliott et al., 2021). Nevertheless, obtaining viable isolates from the vector provides a crucial resource for investigating the maintenance of the pathogen within its natural host and assessing the potential link to human infection (Lee et al., 2025).

In the present study, we successfully isolated three *O. tsutsugamushi* strains from trombiculid mites for the first time, namely Boryong, Kawasaki, and Yonchon. These findings provide direct evidence that viable *O. tsutsugamushi* strains exist within trombiculid mite populations and represent an important foundation for future studies on the ecology and strain diversity of scrub typhus. Notably, these vector-derived isolates serve as a valuable biological resource for direct comparison of genetic and antigenic differences among strains, and they are expected to play a pivotal role in molecular epidemiology research, characterization of pathogen traits, and standardization of reference strains for future investigations.

## 2 Materials and methods

### 2.1 Ethics statement

All animal handling and experimental procedures were approved by the Institutional Biosafety Committee (approval number: KDCA-IBC-2025-007) and the Institutional Animal Care and Use Committee (approval number: KDCA-IACUC-25-002) of the Korea Disease Control and Prevention Agency (KDCA). After obtaining permission under the Wildlife Protection and Management Act, all wild rodents were captured and euthanized in accordance with the approved protocols.

### 2.2 Collection and homogenization of trombiculid mites

Trombiculid mites were collected from wild rodents captured at four sites across the ROK (Northern-Cheorwon, Central-Cheongju, Southern-Geoje and Hapcheon) in November 2025 (**Table 1**). Sherman folding live traps (3 × 3.5 × 9 inches, BioQuip, Gardena, CA, USA), baited with cheese-spread biscuits, were installed at 20 points at 5–10 m intervals in each habitat. The traps were collected the following morning and transported to the laboratory. Captured rodents were euthanized using carbon dioxide (CO_2_) and hung above a Petri dish containing tap water for 24 h to harvest ectoparasites (Kim et al., 2025). Fallen trombiculid mites were carefully recovered from the water surface using a paintbrush, and only live mites were selected for further processing. Trombiculid mites collected from each sampling site were pooled separately, with 50 mites constituting each pooled sample. All 50 mites in each pool were used for homogenate preparation, without subsampling, prior to mouse inoculation. Each pooled sample was washed with 1% sodium hypochlorite (Sigma, St. Louis, MO, USA) and rinsed thoroughly with sterile water (Binetruy et al., 2019; Chaisiri et al., 2023). The washed mites were transferred to a homogenization tube containing phosphate-buffered saline (PBS) and 2.8 mm ceramic (zirconium oxide) beads. The samples were homogenized using a Precellys Evolution tissue homogenizer (Bertin Technologies, Montigny-le-Bretonneux, France) for two cycles of 30 s at 4,000 rpm.

**Table 1:** Isolation and characterization of *O. tsutsugamushi* strains from trombiculid mites.

Strain	Chigger mite collection area	No. of chigger mites inoculated	Days post-inoculation at euthanasia	*Ct value (mouse spleen)	*Ct value (L929 cells, P1)	*Ct value (final L929 cells, P2)	Accession no.
Boryong	Hapcheon (Southern ROK)	50	14	23.94	17.24	19.1	tsutsu_Br9_251118
Kawasaki	Cheongju (Central ROK)		24	21.78	19.96	19.63	tsutsu_Kawa1(CJ):251127
	Geoje (Southern ROK)		20	21.64	20.1	19.05	tsutsu_Kawa8(GJ)_251127
Yonchon	Cheorwon (Northern ROK)		11	22.4	19.64	15.87	tsutsu_Y5_260106

P1 and P2 indicate the first and second sequential passages in L929 cells, respectively. The identity of each *O. tsutsugamushi* strain was confirmed by sequencing of the 56-kDa *tsa* and/or 47-kDa *htrA* genes. *Ct (cycle threshold) values were obtained by real-time quantitative PCR and are presented as descriptive indicators of *O. tsutsugamushi* detection rather than as measures of absolute bacterial burden. Because the analyzed specimens comprised different biological matrices, including mouse spleen tissue and cultured L929 cells, direct quantitative comparisons of Ct values among sample types or strains were not performed. No Ct cut-off value was applied, as reliable and consistent exponential amplification curves were observed in all samples.

### 2.3 Mouse inoculation and euthanasia

Immunodeficient NOD.SCID mice (Koatech, Pyeongtaek, ROK) were purchased and acclimated for one week in individually ventilated iso-cages in an ABL3 facility (24 °C and 60% relative humidity). Following acclimation, 200 µL of trombiculid mite homogenate was administered intraperitoneally (i.p.) using a 1 mL syringe. For negative controls, mice were inoculated with either an equivalent volume of PBS alone or homogenates prepared from trombiculid mite pools confirmed to be PCR-negative for *O. tsutsugamushi*. Mice were monitored daily for clinical signs including piloerection, impaired eye opening, and reduced mobility and all observations were recorded (Soong et al., 2014; Mendell et al., 2017). At approximately 2 weeks post-inoculation, animals exhibiting pronounced clinical symptoms were euthanized using CO_2_ in accordance with institutional ethical guidelines. Following euthanasia, spleens were aseptically collected. One-third of each spleen was used for inoculation, and the remaining tissue was stored at -70 °C.

### 2.4 Isolation and propagation of *O. tsutsugamushi* in L929 cells

Normal L929 cells were cultured at 37 °C with 5% CO_2_ in complete growth medium consisting of DMEM (Gibco, Thermo Fisher Scientific, Waltham, MA, USA) supplemented with 5% fetal bovine serum (FBS; Gibco, Thermo Fisher Scientific) and 1% penicillin-streptomycin (Gibco, Thermo Fisher Scientific) (Jung et al., 2015). For experimental infection with *O. tsutsugamushi*, mouse spleen was passed through a cell strainer and homogenized in infection medium (DMEM, Gibco, Thermo Fisher Scientific), supplemented with 2% FBS and 0.4 µg/mL daunorubicin hydrochloride (Sigma, St. Louis, MO, USA). Daunorubicin hydrochloride was added to limit host-cell proliferation and facilitate *O. tsutsugamushi* propagation (Giengkam et al., 2015). Confluent L929 cells (~90%, 4–6 × 10^6^ cells) were inoculated with 1 mL of the spleen homogenate combined with 7 mL of infection medium and incubated for 2 h at 34 °C with intermittent gentle agitation every 15–20 min to ensure uniform distribution on the cell monolayer. Following the adsorption period, the inoculum was removed, 15 mL of infection medium including 1% penicillin-streptomycin was added (Atwal et al., 2017), and cultures were maintained at 34 °C under 4.5% CO_2_. For serial infection, a portion of the infected cells was passaged onto fresh L929 monolayers in two sequential passages following the same procedure, allowing propagation of *O. tsutsugamushi in vitro*. Subsequently, infected cells were stored at -70 °C to establish cell stocks for downstream applications.

### 2.5 Immunofluorescence assay

Three days post-infection, cytopathic effects (CPE)-like morphological changes, including cell rounding, increased refractility, and focal monolayer disruption were assessed in L929 cell cultures and cells were harvested for IFA (Chang et al., 1990). Infected cells were collected by centrifugation and washed twice with PBS. Cell suspensions were applied onto pre-labeled glass microscope slides, air-dried, and fixed with cold acetone (Sigma) at -20 °C for 10 min. Slides were incubated at 37 °C for 30 min with a human IgG primary antibody (1:400 dilution in PBS), consisting of *O. tsutsugamushi*-positive patient serum provided by the Division of Bacterial Diseases, KDCA. Serum from healthy individuals confirmed to be seronegative for *O. tsutsugamushi* was used as a negative control. After incubation, slides were washed three times, with the first two washes performed using cold PBS and the final wash using distilled water. A fluorophore-conjugated secondary antibody (MP Biomedicals, Solon, OH, USA), diluted 1:400 in PBS containing 0.01% Evans blue (Sigma), was then applied and incubated at 37 °C for 30 min. Following three additional washes, slides were air-dried, mounted with mounting medium (Sigma), and examined under an Axioskop 2 Plus fluorescence microscope (Carl Zeiss, Oberkochen, Germany).

### 2.6 DNA extraction and polymerase chain reaction amplification

Genomic DNA from mite homogenates, spleen and cultured cells was extracted using the G-spin™ Total DNA Extraction Kit (iNtRON Biotechnology, Seoul, ROK), according to the manufacturer’s instructions. For screening and genotyping of *O. tsutsugamushi* at the species level (47-kDa *htrA* gene) and major lineage level (Boryong, Gilliam, Kato, and Karp; 56-kDa *tsa* gene), real-time quantitative PCR (qPCR) was performed using the GeneFinder™ *O. tsutsugamushi* Typing III and Typing IV RealAmp Kits (Osang Healthcare, Anyang, ROK) according to the manufacturer’s instructions. Amplification was carried out on a QuantStudio™ Dx Real-Time PCR System (Thermo Fisher Scientific), and data were analyzed using Design & Analysis Software version 2 (Thermo Fisher Scientific).

Subsequently, conventional PCR targeting the 56-kDa *tsa* gene and the 47-kDa *htrA* gene of *O. tsutsugamushi* was performed to obtain the corresponding nucleotide sequences. Nested PCR for the 56-kDa *tsa* gene was carried out using the LiliF™ TSUTSU Nested PCR Kit (iNtRON Biotechnology, Seongnam, ROK) following previously described methods (Elliott et al., 2019), while PCR amplification of the 47-kDa *htrA* gene was achieved using AccuPower^®^ PCR Premix (BIONEER, Daejeon, ROK) and ProFlex PCR System (Applied Biosystems, Waltham, MA, USA) according to the manufacturer’s instructions. The primers and PCR conditions used for each target gene are summarized in **Table 2**. The amplified PCR products were analyzed by capillary electrophoresis using the QIAxcel Connect system (QIAGEN, Hilden, Germany).

**Table 2:** Primers and nested PCR conditions used for detection of *Orientia tsutsugamushi*.

Pathogen	Target gene	Primer name	Sequence (5’ → 3’)	PCR condition	Size (bp)	Ref.
*Orientia tsutsugamushi*	56-kDa *tsa*	First F	GCA ATA TTG CTA GTG CAA TGT CTG C	94 °C for 5 min; 40 cycles of 94 °C for 30 s, 58 °C for 30 s, and 72 °C for 40 s; 72 °C for 5 min	475	Park et al., 2018
		First R	ATG CAT GCA TGR CGC TKCAA TTT A			
		Second F	ATA GGC CTA TAA GTA TWG CKG ATC G	94 °C for 5 min; 30 cycles of 94 °C for 30 s, 58 °C for 30 s, and 72 °C for 40 s; 72 °C for 5 min		
		Second R	CAT CTA GAY GCA CTA TTA GGC AAA			
	47-kDa *htrA*	Otr47_145F	ACAGGCCAAGATATTGGAAG	95 °C for 2 min; 40 cycles of 94 °C for 30 s, 54 °C for 30 s, and 68 °C for 2 min; 72 °C for 7 min	871	Masakhwe et al., 2018
		Otr47_1780R	AATCGCCTTTAAACTAGATTTACTTATTA			
		Otr47_263F	GTGCTAAGAAARGATGATACTTC			
		Otr47_1133R	ACATTTAACATACCACGACGAAT			

Additionally, to assess the presence of possible intracellular bacterial co-infections during pathogen isolation, nested PCR assays were applied to detect *Rickettsia* spp. (17-kDa, *opmA*, *gltA*), *Anaplasma* spp. (16s rRNA), *Ehrlichia* spp. (16s rRNA), *Borrelia* spp. (*flaB*), and *Coxiella burnetii* (insertion sequence 1111). PCR reactions were carried out using the AccuPower^®^ PCR Premix (**Table 3**).

**Table 3:** Primer sets and nested PCR conditions used for PCR detection of potential bacterial co-infections.

Pathogen	Target gene	Primer name	Sequence (5’ → 3’)	Size (bp)	Ref.
*Rickettsia* spp.	17-kDa	Rr17k.1p	TTTACAAAATTCTAAAAACCAT	539	Yamaguti et al., 1971; Bang et al., 2021
		Rr17k.539n	TCAATTCACAACTTGCCATT		
		Rr17k.90p	GCTCTTGCAACTTCTATGTT	450	
		Rr17k.539n	TCAATTCACAACTTGCCATT		
	*ompA*	R190.70F	ATGGCGAATATTTCTCCAAAAA	634	Bang et al., 2021; Regnery et al., 1991
		RR190.701R	GTTCCGTTAATGGCAGCATCT		
		R190.70F	ATGGCGAATATTTCTCCAAAAA	535	
		RR190.602R	AGTGCAGCATTCGCTCCCCCT		
	*gltA*	RpCS.780p	GACCATGAGCAGAATGCTTCT	479	Ishikura et al., 2003; Reis et al., 2011; Arai et al., 2021; Oh et al., 2009
		RpCS.1258n	ATTGCAAAAAGTACAGTGAACA		
		Rsfg877p	GGGGGCCTGCTCACGGCGG	382	
		Rsfg1258n	ATTGCAAAAAGTACAGTGAACA		
*Anaplasma* spp.	16s rRNA	AE1-F	AAGCTTAACACATGCAAGTCGAA	1406	Seo et al., 2018
		AE1-R	AGTCACTGACCCAACCTTAAATG		
		EE3	GTCGAACGGATTATTCTTTATAGCTTGC	926	Seo et al., 2018; Barlough et al., 1996
		EE4	CCCTTCCGTTAAGAAGGATCTAATCTCC		
*Ehrlichia* spp.	16s rRNA	AE1-F	AAGCTTAACACATGCAAGTCGAA	1406	Oh et al., 2009
		AE1-R	AGTCACTGACCCAACCTTAAATG		
		HE1	CAATTGCTTATAACCTTTTGGTTATAAAT	390	
		HE3	TATAGGTACCGTCATTATCTTCCCTAT		
*Borrelia* spp.	*flaB*	BflaPAD	GATCARGCWCAAYATAACCAWATGCA	451	Sato et al., 1997; Takano et al., 2010
		BflaPDU	AGATTCAAGTCTGTTTTGGAAAGC		
		BflaPBU	GCTGAAGAGCTTGGAATGCAACC	347	
		BflaPCR	TGATCAGTTATCATTCTAATAGCA		
*Coxiella burnetii*	IS1111	IS-F	CAGAGCCACCGTATGAATCAGCTT	959	Enferadi et al., 2023
		IIS-R	TCGGACGTTTATGGGGATGGGTAT		
		ISNF	CACATTGCCGCGTTTACTAATCCC	421	
		ISNR	CACGGCGCTGATCAATGAGATTC		

### 2.7 Sequencing

Nested PCR-amplified products were purified and sequenced by a commercial sequencing service (SolGent, Daejeon, ROK). Sequence chromatograms were visually inspected, trimmed, and assembled into consensus sequences using Chromas software version 2.33 to ensure sequence quality. Species identification was performed by BLASTn analysis against reference sequences available in the GenBank database.

### 2.8 Phylogenetic analysis

Phylogenetic relationships were inferred using the Maximum Likelihood method based on aligned nucleotide sequences. Multiple sequence alignments were conducted using MEGA software version 11.0.13. The robustness of the resulting phylogenetic trees was assessed by bootstrap analysis with 1,000 replicates. The best-fit nucleotide substitution model for each dataset was determined using the model selection function based on the lowest Bayesian information criterion score. The General Time Reversible (GTR) model was applied to the 56-kDa *tsa* gene, whereas the Tamura 3-parameter (T92) model was used for the 47-kDa *htrA* gene.

## 3 Results

### 3.1 Identification of *O. tsutsugamushi* strains for primary isolation

Sequencing revealed that trombiculid mites from Hapcheon carried the Boryong strain, Cheongju and Geoje carried the Kawasaki strain, and Cheorwon carried the Yonchon strain. These strains were selected as candidates for pathogen isolation. Only mice inoculated with these identified strains through *in vivo* passage were included in subsequent observations and molecular analyses.

### 3.2 Confirmation of *O. tsutsugamushi* isolation in NOD.SCID mice

The qPCR analysis confirmed the presence of *O. tsutsugamushi* and revealed low Ct values ranging from 21.64 to 23.94 in all spleen homogenate samples (**Table 1**). In addition, sequencing results demonstrated that the pathogen isolated from mouse spleens was genetically identical to the strain detected in the corresponding trombiculid mite homogenates used for inoculation. These findings indicate that trombiculid mite-derived *O. tsutsugamushi* was successfully isolated and maintained in NOD.SCID mice. In contrast, mice inoculated with PCR-negative trombiculid mite homogenates, as well as PBS-inoculated negative-control mice, remained PCR-negative for *O. tsutsugamushi* throughout the observation period.

### 3.3 Establishment of *O. tsutsugamushi* isolates in L929 cell cultures

CPE-like morphological changes were observed in infected L929 cells at 3 days post-infection following inoculation with spleen homogenates obtained from infected mice, whereas no such effects were observed in uninfected control cells (**Figures 1A, B**). IFA revealed distinct green fluorescent signals localized in the perinuclear region of infected cells, while no specific fluorescence was observed in negative control cells. qPCR analysis of infected cells showed low Ct values between 17.24 and 20.1 (**Table 1**), confirming active intracellular replication of *O. tsutsugamushi*.

**Figure 1:**
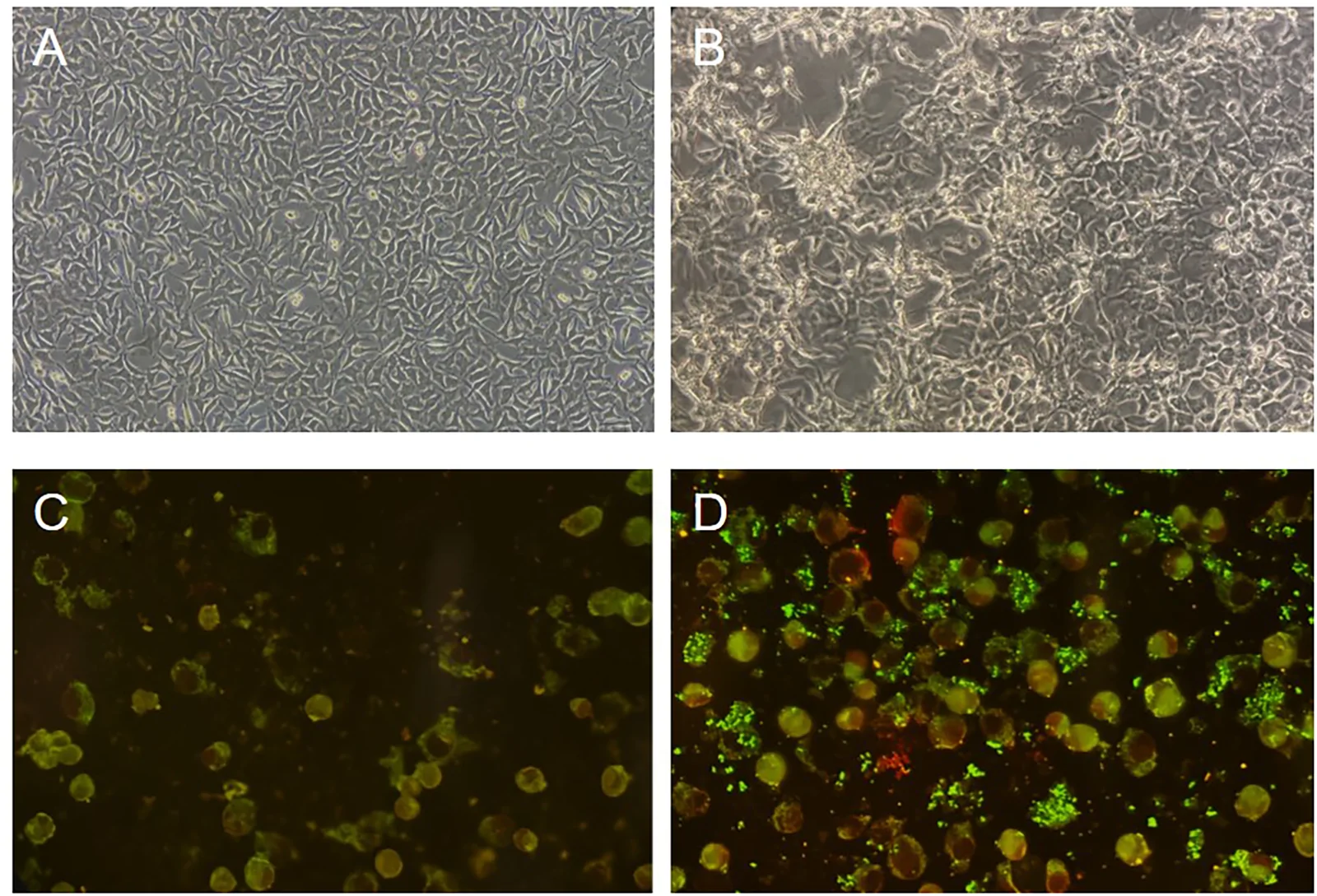
Optical and fluorescence microscopic observation of L929 cells infected with *Orientia tsutsugamushi*. **(A)** Optical microscopy image of the L929 cell monolayer before inoculation (×200). **(B)** Optical microscopy image of L929 cells showing CPE-like morphological changes at 3 days post-infection (×200). **(C)** Immunofluorescence assay (IFA) of infected L929 cells using negative serum as a control (×400). **(D)** IFA of infected L929 cells using positive serum, showing green fluorescent dots representing intracellular *O. tsutsugamushi* (×400).

### 3.4 Stability of *O. tsutsugamushi* isolates during serial passage

L929 cells infected with *O. tsutsugamushi* were serially passaged twice. Following the final passage, IFA revealed consistent fluorescent signals, and qPCR analysis of the same samples showed persistently low Ct values ranging from 15.87 to 19.63 (**Table 1**). Nested PCR targeting the 56-kDa *tsa* and 47-kDa *htrA* genes (**Figure 2**), followed by sequencing, confirmed that the pathogen isolated from consecutive passages was identical to the strains detected in the original trombiculid mite homogenates and mouse spleens. Phylogenetic analysis based on the sequenced gene fragments further demonstrated that the isolates clustered within the established lineages of *O. tsutsugamushi*, specifically corresponding to the Boryong, Kawasaki, and Yonchon strains (**Figure 3**). These results demonstrate that the isolates were stably maintained and propagated during serial *in vitro* passage, while retaining the genetic characteristics of the original strains.

**Figure 2:**
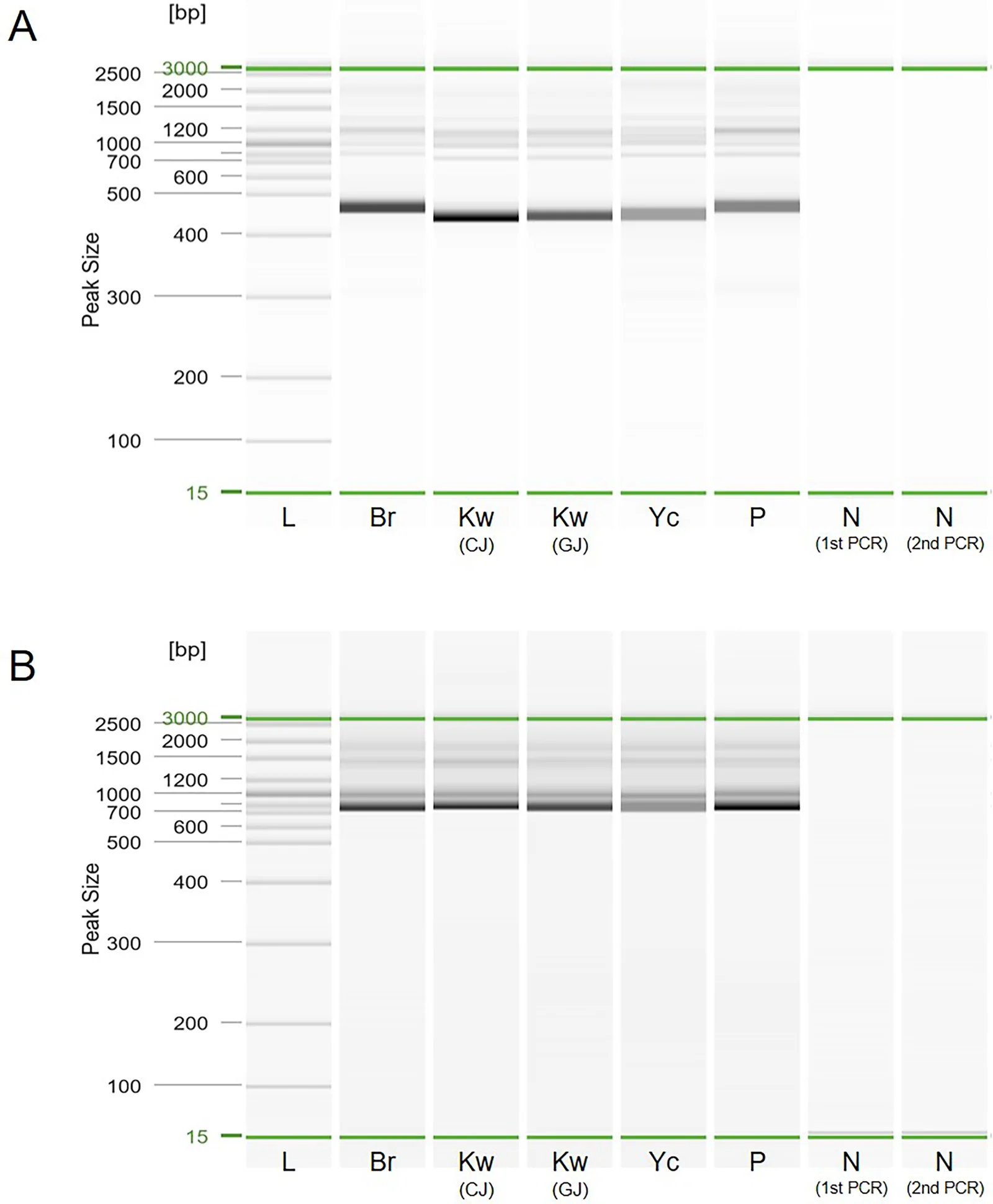
PCR amplification of *Orientia tsutsugamushi* gene fragments from L929 mouse fibroblast cells infected with three isolates obtained from trombiculid mites. PCR electrophoretic profiles of the final isolates are shown. **(A)** Amplification of the 56-kDa *tsa* gene fragment (475 bp). **(B)** Amplification of the 47-kDa *htrA* gene fragment (871 bp). Lanes represent: L, DNA ladder (15–3,000 bp; Thermo Scientific); Br, *O. tsutsugamushi* Boryong isolate; Kw (CJ) and Kw (GJ), Kawasaki isolates from Cheongju and Geoje, respectively; Yc, Yonchon isolate; P, positive control using Boryong genomic DNA; N (1st PCR) and N (2nd PCR), negative controls using nuclease-free water.

**Figure 3:**
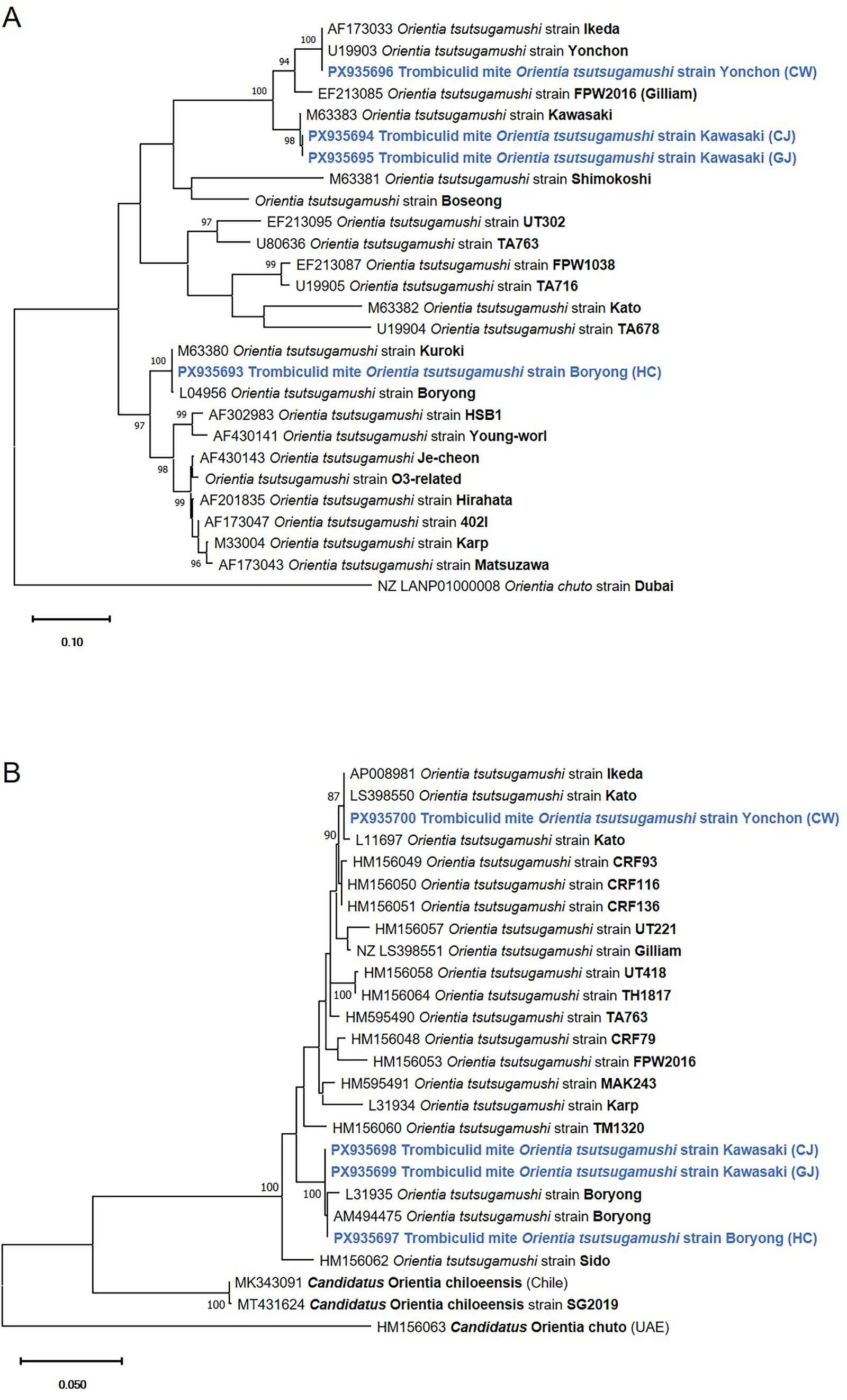
Phylogenetic trees of *Orientia tsutsugamushi* based on nucleotide sequences of the 56-kDa *tsa* gene **(A)** and the 47-kDa *htrA* gene **(B)**. The isolates analyzed in this study clustered with the Boryong, Kawasaki, and Yonchon strains. Trees were constructed using the Maximum Likelihood method with 1,000 bootstrap replicates. Bootstrap values are shown at the nodes and scale bars indicate nucleotide substitutions per site. An 80% cutoff value was applied to the consensus tree. The sequences generated in this study are shown in blue, and their GenBank accession numbers are PX935693–PX935700.

### 3.5 Quality assessment and preservation of *O. tsutsugamushi* isolates

To confirm the absence of bacterial co-infection, all isolates tested negative for intracellular bacteria including *Rickettsia* spp., *Anaplasma* spp., *Ehrlichia* spp., *Borrelia* spp., and *C. burnetii*. The verified *O. tsutsugamushi* isolates—Boryong, Kawasaki, and Yonchon—were cryopreserved as infected cell stocks at -70 °C and subsequently deposited in the National Culture Collection for Pathogens (NCCP; Cheongju, ROK) under the accession numbers tsutsu_Br9_251118, tsutsu_Kawa1(CJ)_251127, tsutsu_Kawa8(GJ)_251127, and tsutsu_Y5_260106, respectively (**Table 1**).

## 4 Discussion

This study demonstrates that viable *O. tsutsugamushi* can be successfully recovered from field-collected trombiculid mites and subsequently amplified and stably propagated using a combined *in vivo*–*in vitro* system (Rodkvamtook et al., 2011; Giengkam et al., 2015). Trombiculid mites play a central role in the transmission of scrub typhus and serve as natural reservoirs, maintaining the pathogen through transstadial and transovarial transmission (Xu et al., 2017). Therefore, direct investigation of *O. tsutsugamushi* in trombiculid mites is important for understanding the natural maintenance and transmission of the pathogen (Elliott et al., 2019). Most previous studies have primarily relied on PCR-based detection of *O. tsutsugamushi* DNA in mites, which provides valuable information on pathogen distribution but does not demonstrate the presence of viable organisms (Zhang et al., 2013; Prakash et al., 2022). In contrast, the present study successfully isolated viable *O. tsutsugamushi* strains from trombiculid mites collected from geographically distinct regions of the ROK. These findings extend previous molecular surveillance by confirming that *O. tsutsugamushi* detected in natural mite populations can exist as viable organisms and provide biological isolates that can be used for further genetic and phenotypic characterization.

The Boryong, Kawasaki, and Yonchon lineages isolated in this study correspond to *O. tsutsugamushi* lineages previously reported in human scrub typhus cases and vector surveillance studies in the ROK. In particular, the Boryong strain has been frequently detected in human patients (Park et al., 2010) and has also been identified in trombiculid mites and wild rodents in southern regions of the ROK (Ree et al., 2001; Seo et al., 2021; Lee et al., 2025). Although the Kawasaki strain has been reported at a relatively lower frequency, it has been identified in both patients and vectors from central regions and is recognized as one of the circulating lineages in the ROK (Park et al., 2010; Jeong et al., 2012; Seo et al., 2021). The Yonchon strain has also been reported in human patients (Seong et al., 1997; Kim et al., 2008) and in trombiculid mites collected from northern regions of the ROK (Lee et al., 2025). However, establishing a direct strain-level epidemiological relationship between vector-derived isolates and human infections would require comparative analyses of corresponding human- and vector-derived isolates. Future whole-genome sequencing of these mite-derived isolates, followed by comparison with clinical isolates obtained from human scrub typhus cases during the same period, could help clarify strain-level epidemiological links between vector populations and human infections.

A total of 50 mice were used in the *in vivo* isolation experiments. Except for the negative-control animals, which received PBS alone, all mice were inoculated intraperitoneally with homogenates prepared from pooled field-collected trombiculid mites. During the first week after inoculation, most mice remained active, exhibited apparently normal feeding and resting behavior, and showed no obvious clinical abnormalities. However, beginning at approximately 7 days post-inoculation, clinical signs developed in a subset of the inoculated mice, corresponding to approximately 30% of the animals. The absence of overt clinical signs in some mice may have been attributable to several factors. Because each inoculum was prepared from a pool of approximately 50 field-collected trombiculid mites, the proportion of *O. tsutsugamushi*-infected mites likely varied among pools, ranging from pools containing no infected mites to those containing multiple infected mites. In addition, partial loss of bacterial viability during processing and preparation of the mite homogenates may have reduced the effective infectious dose. Variability in the effective delivery of the inoculum during intraperitoneal injection may also have contributed to differences in the establishment of infection among individual animals. In mice with clinical signs, the initial manifestations included reduced mobility, piloerection, and tremor, which progressively became more pronounced during the second week after inoculation. At the later stage of infection, severely affected mice exhibited marked lethargy and impaired eye opening. Marked and progressive clinical deterioration was considered indicative of sufficiently progressed infection and was used to determine that the humane endpoint had been reached. These mice were subsequently euthanized. Accordingly, the timing of euthanasia was determined based on the clinical condition of each individual animal rather than at a predetermined fixed time point after inoculation.

Following euthanasia, the spleen was selected as the primary tissue for subsequent isolation and molecular detection. Although *O. tsutsugamushi* can disseminate systemically and may also be detected in organs such as the liver, lung, and kidney (Keller et al., 2014; Shelite et al., 2014; Soong et al., 2016), the objective of the present study was to recover viable organisms rather than to characterize tissue tropism or organ-specific bacterial burden. The spleen provides a practical cell-rich tissue for recovery of systemic intracellular bacteria and allowed sufficient tissue material to be obtained for both molecular detection and subsequent inoculation into cell culture. Consequently, bacterial loads in other organs were not quantitatively evaluated in this study. This limitation means that the present data should not be interpreted as evidence that the spleen is the preferential target organ of the recovered isolates. Comparative quantification of bacterial loads across multiple organs using qPCR or other quantitative approaches will be required in future studies to determine tissue distribution and potential strain-dependent differences in tropism.

The relatively early appearance of CPE-like morphological changes within 3 days after inoculation may suggest that a substantial amount of viable *O. tsutsugamushi* was introduced into the L929 cell cultures. Here, the term “CPE-like” refers to morphological alterations associated with intracellular *O. tsutsugamushi* infection rather than classical virus-induced CPE. In the present study, 1 mL of spleen homogenate was used as the inoculum because the primary objective was to maximize the probability of recovering viable organisms from field-derived material, in which the initial bacterial burden was unknown and expected to vary among samples. Thus, the inoculation procedure was designed primarily for successful isolation and propagation rather than for standardized comparison of infectivity or growth kinetics among strains.

In this study, the presence of viable intracellular *O. tsutsugamushi* was primarily verified by IFA following the observation of CPE-like morphological changes. However, quantitative comparisons of pathogenicity or serological responses based on antibody titers were not performed. IFA was considered sufficient for the study objectives, as it allows direct visualization of intracellular pathogens and their localization, which is adequate for assessing strain maintenance (**Figures 1C, D**) (Kim et al., 2010, 2021). Additionally, qPCR was used to evaluate the successful isolation of *O. tsutsugamushi* in the host. Because this analysis was not intended for absolute quantification, bacterial loads are reported as Ct values rather than copy numbers. This approach provided a reliable indication of bacterial replication and persistence within the host, adequately fulfilling the study objectives (Jian et al., 2020; Bouzid et al., 2021). The qPCR analyses showed consistently low Ct values for the *O. tsutsugamushi* species-specific target across all samples, indicating robust bacterial replication during mouse passage and subsequent cell culture and confirming the functionality of the isolated strains (**Table 1**). Notably, strain-specific qPCR assays for the Yonchon strain revealed additional amplification signals corresponding to the Gilliam lineage (data not shown), consistent with previous reports that the Yonchon strain is classified as Gilliam-related (Lee et al., 2025). However, nested PCR and sequence analysis confirmed that each isolate represented a single dominant lineage (**Figure 3**), suggesting genetic variability within the 56-kDa type-specific antigen gene rather than mixed infection. Importantly, these results confirm that the pathogen isolation process reliably produced pure and viable strains suitable for subsequent downstream studies.

Several experimental strategies were critical for the successful isolation and stable maintenance of these isolates. Surface washing of trombiculid mites with sodium hypochlorite was an essential step, as it effectively removed surface-associated bacteria while preserving the internal microbiome and microbial viability (Binetruy et al., 2019; Chaisiri et al., 2023). This procedure minimized external microbial contamination without affecting the intracellular pathogen. The use of immunodeficient NOD.SCID mice was also a critical factor in the successful isolation of *O. tsutsugamushi* (Jiang et al., 2018). Owing to the absence of adaptive immune responses, this model provided a permissive environment that enabled efficient bacterial amplification from trombiculid mite homogenates presumed to contain low bacterial loads (Bente et al., 2005). Another major strength of this study was the systematic exclusion of co-infection with representative tick- and trombiculid-borne intracellular bacteria including *Rickettsia* spp., *Anaplasma* spp., *Ehrlichia* spp., *Borrelia* spp., and *C. burnetii* (Vaca et al., 2022; Oh et al., 2024). In particular, *Rickettsia* species have intracellular lifestyles similar to that of *O. tsutsugamushi* and cannot be readily distinguished based solely on CPE-like morphological changes or IFA findings, making exclusion of co-infection essential (Bhandari et al., 2022). Although trombiculid mites in natural environments are likely to harbor diverse bacterial communities, the absence of detectable co-infections in the final isolates suggests that passage through NOD.SCID mice functioned as a biological filter, allowing a single *O. tsutsugamushi* strain to dominantly replicate. In addition, *O. tsutsugamushi* is intrinsically resistant to penicillin-streptomycin, as reported in prior studies (Atwal et al., 2017; Tantibhedhyangkul et al., 2019). Thus, the use of infection medium containing these antibiotics after the adsorption phase suppressed extracellular bacterial growth without impacting the replication of the intracellular pathogen. Together, these carefully optimized conditions, including the use of sodium hypochlorite surface washing, immunodeficient mice, and antibiotic-supplemented culture medium, were essential for the successful establishment and stable maintenance of the isolates, allowing reliable propagation of *O. tsutsugamushi* without interference from external contaminants.

Despite these strengths, this study has several limitations. First, species-level identification of individual trombiculid mites involved in pathogen isolation was not possible. Trombiculid mites were processed in pools of approximately 50 individuals per sample prior to inoculation into NOD.SCID mice (**Table 1**), which precluded the identification of the specific mite species harboring *O. tsutsugamushi*. Consequently, direct associations between the isolated strains and particular trombiculid mite species could not be established, limiting detailed interpretations of vector-strain specificity and ecological transmission dynamics. In addition, pooling may have masked intra-pool strain diversity, because multiple *O. tsutsugamushi* strains could have been present within a single mite pool. The use of pooled samples likely favored the isolation of a single dominant *O. tsutsugamushi* strain from each sample, potentially overlooking minor co-circulating strains present at lower abundance within the same pool. However, given that the primary objective of this study was to directly isolate and stably establish viable *O. tsutsugamushi* strains from natural trombiculid mite populations, the impact of these limitations on the main conclusions of the study is considered limited. Future studies incorporating individual mite-based screening together with morphological or molecular species identification will be required to more precisely elucidate vector competence, strain diversity, and the ecological relationships between specific trombiculid mite species and *O. tsutsugamushi* lineages.

Second, phenotypic differences among the different *O. tsutsugamushi* strains were not systematically compared or characterized in the present study. In infected mice, common clinical signs, including piloerection, impaired eye opening, and reduced mobility, were observed regardless of the strain. However, the onset and progression of these clinical signs varied slightly among strains, resulting in differences of several days in the timing of euthanasia, as shown in **Table 1**. Because these temporal differences were not quantitatively evaluated, it remains unclear whether the observed differences in the onset and progression of clinical signs reflect genuine strain-specific biological variation. Similarly, no clearly distinguishable differences in CPE-like morphological changes were observed among the strains during infection of L929 cells. Therefore, the *in vivo* clinical signs and *in vitro* cellular changes observed in this study should be interpreted primarily as common features of *O. tsutsugamushi* infection under the experimental conditions used, rather than as strain-specific phenotypes. Further studies incorporating quantitative comparisons of bacterial growth kinetics, the onset and severity of clinical signs, tissue bacterial loads, histopathological changes, and cellular infection characteristics will be necessary to more clearly define the pathogenic and phenotypic characteristics of individual strains and identify potential strain-specific differences.

Third, although the use of immunodeficient NOD.SCID mice facilitated efficient isolation and amplification of *O. tsutsugamushi*, it limited the ability to quantitatively assess host immune responses and pathogenicity. Because NOD.SCID mice are severely deficient in adaptive immune responses, the infection patterns observed in this model may not fully reflect inflammatory responses, immune-evasion mechanisms, or strain-specific host–pathogen interactions in immunocompetent hosts (Nagano et al., 1996). Therefore, additional infection studies using immunocompetent animal models and incorporating immunological parameters, such as inflammatory cytokine responses, will be required to more comprehensively characterize these aspects of *O. tsutsugamushi* infection (Soong et al., 2016; Mendell et al., 2017). Despite these limitations, the vector-derived isolates established in this study represent valuable biological resources for molecular and functional studies of scrub typhus.

In conclusion, this study demonstrates the successful recovery and establishment of *O. tsutsugamushi* strains from trombiculid mites, the vectors of scrub typhus. These findings enhance our understanding of the ecology of *O. tsutsugamushi* and provide an important experimental foundation for future studies, including molecular epidemiology, diagnostic development, vaccine candidate evaluation, and pathogen characterization. The isolates established in this study were deposited in a biological resource repository, thereby ensuring their accessibility as standardized research resources. Future studies should further characterize these isolates using high-resolution genomic approaches, such as next-generation sequencing, to assess strain purity, confirm genomic integrity, and identify potential genomic differences among the isolates. In addition, comparative genomic and antigenic analyses with clinically derived strains, together with more detailed phenotypic and pathogenicity studies, will be important for clarifying strain-specific characteristics and the molecular determinants associated with pathogenicity and diagnostic performance. Collectively, the vector-derived isolates generated and deposited in this study provide valuable biological resources for a broad range of future investigations.

## Data Availability

The datasets presented in this study can be found in online repositories. The names of the repository/repositories and accession number(s) can be found in the article/supplementary material.
